# Relationships between upper- and lower-limb strength and power with ball velocity in university handball players

**DOI:** 10.3389/fspor.2026.1864528

**Published:** 2026-09-08

**Authors:** Maraysa Spagnollo Da Silva, Júlia Araujo Pavan, Enrico Fuini Puggina

**Affiliations:** Physical Performance and Sports Training Research Group, School of Physical Education and Sport of Ribeirão Preto (EEFERP), University of São Paulo (USP), Ribeirão Preto, Brazil

**Keywords:** ballistic push-up, sports performance, strength and power, team sports, throwing velocity

## Abstract

**Introduction:**

Throwing velocity is a key determinant of offensive success in handball. While the relationship between muscle strength and ball velocity is well-documented, the effectiveness of practical functional indicators, such as the Ballistic Push-up (BPU), remains under-investigated in this sport. This study aimed to analyze the relationship between various upper- and lower-limb strength and power assessments and ball velocity in the standing throw (ST) and the jumping throw with progression (JT).

**Methods:**

Twelve university athletes (6 males, 6 females; 22.8 ± 3.7 years) completed a testing battery across six sessions, including: maximal dynamic strength (1RM) in the bench press, bench press power at three intensities (30%, 60%, and 90% of 1RM), seated medicine ball (MB) throw, BPU variations (Standard, Kneeling, and Drop-fall), and the countermovement jump (CMJ). Ball velocity was quantified via 2D kinematic analysis (240 fps) using Kinovea software.

**Results:**

Strong to very strong correlations were observed between bench press variables and throwing performance. The JT showed the most robust associations with 1RM (*ρ* = 0.842) and peak power at 60% of 1RM (*ρ* = 0.846). Conversely, the ST correlated more significantly with light-load variables at 30% of 1RM (mean acceleration: *ρ* = 0.748). The Standard Ballistic Push Up (BPU) and CMJ emerged as significant predictors specifically for the JT (*ρ* = 0.734 and *ρ* = 0.748, respectively). Unexpectedly, the seated MB throw showed negative correlations with ball velocity.

**Conclusion:**

The findings suggest that JT performance depends on force production at moderate-to-high intensities, while ST is more closely associated with high-velocity, low-resistance capacities. The bench press and BPU are valid and practical functional indicators for monitoring and individualizing strength and power training in handball athletes.

## 1 Introduction

Olympic handball first appeared at Berlin 1936, then was no longer part of the Olympic program after World War II, and returned permanently for men in Munich 1972; women were added in 1976 (1, 2). Since its reintroduction in the Olympic Games in 1972, handball has consolidated itself as a high-intensity invasion team sport with widely investigated physical and physiological demands (3). The game is characterized by a constant cycle of high-intensity intermittent actions, such as sprinting, changing direction, jumping, and physical contact (4–6). Within this dynamic context, throwing performance plays a key role in offensive success. Greater throwing velocity has been associated with enhanced offensive effectiveness, as faster ball speeds reduce the time available for goalkeeper responses and may increase the likelihood of successful shot outcomes (7). Previous biomechanical and performance studies have identified throwing velocity as an important determinant of handball performance (8–10).

Ball velocity during handball throws results from a complex kinetic chain in which force is generated in the lower limbs, transferred through the trunk, and ultimately applied through the throwing arm (11). Consequently, both upper- and lower-limb muscle strength and power can be considered as fundamental physical determinants of throwing performance. Previous literature has established that resistance training correlates positively with throwing speed (12, 13), however, throwing is an explosive action that is highly dependent on the capacity of the limbs for rapid force production. In this context the specificity of the assessment methods remains a topic of debate (14).

Research has extensively explored physical tests as predictors of ball velocity in handball, highlighting the medicine ball (MB) throw for its high correlation with performance. However, these correlations are sensitive to implement weight and task constraints. Rivilla-García et al. (15) observed that correlations decreased from *ρ* = 0.904 (800 g) and *ρ* = 0.781 (3 kg) without opposition to *ρ* = 0.647 and *ρ* = 0.743 with a goalkeeper, respectively. Additionally, Debanne et al. (14) reported a robust correlation (*ρ* = 0.800) using a 2 kg kneeling MB throw. While maximum strength, often assessed via 1RM bench press, is considered a foundational capacity, as training-induced strength gains improve throwing velocity (12), its direct association with ball velocity is moderate (*ρ* = 0.55), indicating it is not the sole determinant of throwing power. In addition to traditional resistance training using fixed loads, velocity-based methods employing individualized loads have also demonstrated improvements in maximal strength and throwing velocity in handball athletes (16). Furthermore, sport-specific training and assessment methods, including the bench press, ballistic training, elastic resistance exercises, and plyometric training, have been shown to transfer to throwing performance by enhancing power production through movement patterns that closely resemble the sport-specific actions performed during handball throws (17–20). Furthermore, lower-limbs power tests like CMJ demonstrate low to moderate correlations with standing throw (ST) and jump throw (JT) (21).

Despite this evidence, methodological variability among strength and power assessments creates a gap in the identification of practical and sport-specific evaluation tools. The Ballistic Push-Up (BPU) has emerged as a promising functional alternative and has been validated as a measure of upper-limb power in other populations (22). Its biomechanical similarity to the throwing kinetic chain and the use of body mass as the external resistance make it particularly attractive for sport-specific assessment. However, because BPU performance is inherently influenced by body mass, differences in body composition may affect the mechanical output obtained during the test. Moreover, its predictive value for handball-specific performance has not yet been established. Therefore, the present study aimed to investigate the relationships between different strength and power assessment methods, including the BPU, and ball velocity during different handball throwing techniques. We hypothesized that functional power-based tests would demonstrate stronger associations with ball velocity than isolated maximal strength assessments, as their neuromuscular characteristics more closely resemble the specific demands of the throwing task.

## 2 Materials and methods

### 2.1 Study design

This cross-sectional study was conducted over six visits to the Cineanthropometry and Human Performance Laboratory (LaCiDH) of the School of Physical Education and Sport of Ribeirão Preto (EEFERP – University of São Paulo). The research protocol was approved by the local Ethics Committee (School of Physical Education and Sport of Ribeirão Preto – São Paulo, Brazil. Protocol: CAAE – 78852817.2.0000.5659), and all participants provided written informed consent before enrollment. The six-visit protocol was designed to reduce the duration of each testing session while accommodating the participants' academic schedules. Because the testing battery comprised several physically demanding assessments with similar movement patterns, the protocol was distributed across multiple visits to minimize fatigue and ensure consistent performance throughout the evaluation period.

The first two visits were dedicated exclusively to familiarization with all physical and sport-specific testing procedures. To facilitate participants' schedules, familiarization was completed over two sessions. During the first session, participants performed the bench press tests, including the one-repetition maximum (1RM) and bench press power assessments. During the second session, participants completed the medicine ball throw, ballistic push-up (BPU), and handball throwing tests. The remaining four visits were used for data collection, which was completed within a maximum period of four weeks. During the final two testing visits, the order of the power and throwing assessments was randomized using a web-based randomization tool to minimize cumulative fatigue and acute learning effects.

Before each testing session, participants completed a standardized warm-up consisting of 5 min of cycling on a stationary ergometer at a self-selected intensity, followed by dynamic upper-limb mobility and shoulder activation exercises. Owing to the participants' academic and personal commitments, it was not possible to standardize the testing time across individuals. However, to minimize the potential influence of circadian variation, each participant completed all evaluation sessions at approximately the same time of day.

### 2.2 Participants

Sample size was calculated *a priori* based on a study published about using anthropometric variables and isotonic tests to predict the ball velocity in handball (14). The required sample size was estimated *a priori* using an expected correlation coefficient of 0.80, a significance level (*α*) of 0.05, and a statistical power of 80%. Assuming a 15% dropout rate, a total sample of 12 university handball players was required. The sample comprised 12 Brazilian university-level and regional handball players [tiered as Tier 2: Trained/Developmental (23)], equally divided between males (*n* = 6) and females (*n* = 6). Inclusion criteria required athletes to participate in at least one handball training session per week and to have been engaged in a resistance training program for a minimum of four weeks. Participants with any musculoskeletal injury within the three months preceding data collection were excluded from the study. To ensure compliance with this eligibility criterion, all participants signed a declaration confirming that they had not sustained any musculoskeletal injuries during this period before participating in the study.

### 2.3 Procedures and measures

#### 2.3.1 Anthropometry and body composition

Body mass, height, and arm span were measured using a digital scale, a measuring tape, and a stadiometer, respectively. Body mass was assessed using the same digital scale for all participants. Arm span was measured with participants standing with their back against a wall and both arms fully extended horizontally. The tips of the middle fingers were marked on the wall, and the distance between the two marks was measured using a measuring tape. Height was assessed with participants standing barefoot against the wall with their feet together in an upright position. The highest point of the head was marked on the wall, and the vertical distance from the floor to the mark was measured using the stadiometer. The arm span-to-height ratio was calculated for sample characterization. Skeletal muscle mass, body mass index, and body fat percentage were estimated using a tetrapolar bioelectrical impedance analyzer (BIA1011AF, AF Sanny, Brazil). Body composition was assessed using a bioelectrical impedance analyzer following the manufacturer's recommended protocol. Participants were instructed to fast for at least 4 h before the assessment, refrain from physical activity during the 24 h preceding the test, and empty their bladder immediately before the measurement. The device was calibrated before each assessment according to the manufacturer's instructions. Electrodes were placed on the right hand and right foot as recommended by the manufacturer, with two electrodes positioned on each limb: one placed proximally near the wrist and ankle joints, respectively, and the second positioned at the base of the middle finger and the base of the second toe.

#### 2.3.2 Upper-limb strength and power assessments

Upper-body maximal strength and muscle power were assessed during the bench press exercise using a 14 kg semi-Olympic barbell. To minimize the number of maximal attempts and reduce acute neuromuscular fatigue in these non-professional athletes, baseline one-repetition maximum (1RM) loads were estimated during the familiarization session using the Brzycki equation (24). During the experimental session, maximal dynamic strength was determined using the 1RM test, whereas upper-body muscle power was evaluated on a separate day using a linear position transducer (Peak Power®, CEFISE, Nova Odessa, Brazil). The transducer operates with an internal sampling rate of 50 kHz, it was individually aligned with each participant's shoulder axis to ensure a vertical cable trajectory throughout the movement. Participants performed three submaximal sets at 30%, 60%, and 90% of their 1RM (8, 6, and 1 repetition, respectively) and were instructed to execute the concentric phase with maximal intentional velocity. Valid repetitions required the barbell to touch the chest at the end of the eccentric phase and to reach full elbow extension at the end of the concentric phase. The 1RM assessment was performed using a controlled cadence (approximately 1 s for both the eccentric and concentric phases). Peak power (W), mean force (*N*), mean velocity (m·s⁻^1^), and mean acceleration (m·s⁻^2^) were obtained from the linear position transducer. Rest intervals were standardized at 3 min between warm-up sets and 5 min between maximal attempts and between the strength and power assessments.

Additionally, upper-limb explosive power was assessed using the Medicine Ball (MB) throw and the Ballistic Push-Up (BPU) test. For the MB throw, participants performed the test from a seated position with their back fully supported against a wall with both hands, using a 1 kg medicine ball for females and a 3 kg medicine ball for males. Three maximal attempts were performed, each separated by 30 s of passive recovery. Throwing distance was measured from the starting position to the first point of contact of the ball with the floor, and the longest distance achieved was used for subsequent analyses. The BPU test was performed on a contact mat system (Jump System Pro®, CEFISE, Nova Odessa, Brazil) and included three variations: Standard Countermovement Push-up (SCP), Kneeling Countermovement Push-up (KCP), and Drop-Fall Push-up (DFP) (22). Participants completed three maximal trials of each variation with 30 s of passive recovery between repetitions. Flight time, jump height, absolute power (W), and relative power (W·kg⁻^1^) were automatically calculated by the contact mat system based on flight time.

#### 2.3.3 Lower-limb power assessment

Lower-limb power was assessed using the countermovement jump (CMJ) test on a contact mat system (Jump System Pro®, CEFISE, Nova Odessa, Brazil). The CMJ assessment was integrated into the testing battery and performed during the fifth visit in a randomized order. As all participants completed the standardized general warm-up protocol at the beginning of the session, no additional warm-up was performed before the jump trials. Participants executed three maximal jumps with their hands maintained on their hips, separated by 30 s of passive recovery. They were instructed to perform a rapid countermovement to a self-selected depth, followed immediately by a maximal explosive vertical jump upon the evaluator's signal and they also had to land with theis knees extended to guarantee the right time flight. Jump height was calculated from flight time by the contact mat system, while absolute power (W) and relative power (W·kg⁻^1^) were automatically computed after entering each participant's body mass measured on the testing day. The best of the three trials was used for subsequent analyses.

#### 2.3.4 Handball throwing performance

Throwing kinematics were analyzed under two sport-specific conditions: a standing throw (ST) performed from the 7-m line and a jump throw (JT) with a three-step approach performed from the 9-m line. These distances were selected to replicate two common game situations: the 7-m penalty throw and the jump throw typically executed near the 9-m line to overcome the defensive block (25). For the JT, the 9-m line served as a reference to ensure that participants initiated their jump from a consistent location while maintaining the ecological validity of the movement. Participants used official handballs according to their sex category and were allowed to use resin if they routinely did so during training and competition.

All throws were recorded at 240 Hz using a high-speed camera (GoPro Hero 9 Black; 1080p resolution). Ball kinematics were analyzed using Kinovea® software (version 2023.1.2) (26). The frame in which the ball was visually identified as completely leaving the participant's hand was defined as the initial frame, and the second subsequent frame was used as the final frame for calculating the ball's initial release velocity and acceleration from its two-dimensional displacement. The software was calibrated according to the camera frame rate and using a known court distance (the distance between the goal line and the 7-m line) as the spatial reference. The camera was positioned on the sideline, 8 m from the goal line, providing a perpendicular view of the throwing plane to minimize perspective errors during video analysis.

### 2.4 Statistical analysis

Data were analyzed using JASP (version 0.17.3.0, Amsterdam, The Netherlands). The sample size (*n* = 12) was determined *a priori* based on an expected correlation coefficient of 0.80 reported on previous study (14), assuming *α* = 0.05 and a statistical power (1−*β*) of 0.80. An additional *post-hoc* power analysis was performed using G*Power (version 3.1.9.7; Heinrich-Heine-Universität Düsseldorf, Germany) as a descriptive measure of the achieved study sensitivity.

Data normality was formally evaluated using the Shapiro–Wilk test. Because several continuous variables displayed non-normal distributions, and considering the small sample size (*n* = 12), non-parametric statistics were uniformly applied to ensure robust statistical inference. Descriptive statistics are reported as mean standard deviation (SD) for normal demographic descriptors and as median and interquartile range (IQR) for performance variables. To determine the strength and direction of bivariate relationships between physical performance measures and ball throwing velocity (ST and JT), Spearman's rank correlation coefficient (rho) was utilized as the standardized effect size. The magnitude of correlation coefficients was interpreted according to Sawilowsky (27): as trivial (<0.10), low (0.10–0.29), moderate (0.30–0.49), strong (0.50–0.69), very strong (0.70–0.89), or nearly perfect (0.90–1.00). Two-tailed statistical significance was set *a priori* at *p* < 0.05 for all analyses.

## 3 Results

The general anthropometric characteristics of the sample (*n* = 12) are presented in Table 1. Although the inclusion criteria required a minimum of four weeks of prior physical training, the selected participants reported being engaged in regular training for at least 4 weeks.

**Table 1:** Descriptive characteristics of the sample (*n* = 12).

Variable	Mean ± SD
Age (years)	22.8 ± 3.7
Body mass (kg)	74.067 ± 12.39
Skeletal muscle mass (kg)	31.118 ± 8.85
Body mass index (kg/m^2^)	25.098 ± 2.26
Arm span (m)	1.725 ± 0.10
Height (m)	1.708 ± 0.10
Arm span/height ratio	1.010 ± 0.02
Body fat (%)	24.231 ± 6.30

The data from Table 2 is the most prominent finding, a very strong and significant correlation between maximal dynamic strength (1RM) and JT velocity. Similarly, peak power at 60% of 1RM demonstrated a very strong correlation with JT. Another relevant outcome was the mean acceleration at 30% of 1RM, which correlated strongly with both the ST and JT, indicating that the ability to rapidly accelerate a light load is significantly associated with ball velocity.

**Table 2:** Descriptive and correlation data for bench press strength and power variables**.**

Variable	Median ± IQR	Spearman's *ρ* (ST)	Spearman's *ρ* (JT)
1RM (kg)	70.00 ± 37.25	0.688*	0.842*
Peak power – 30% 1RM (W)	466.60 ± 43.80	0.580	0.692*
Mean acceleration – 30% 1RM (m/s^2^)	6.01 ± 1.59	0.748*	0.755*
Mean force – 30% 1RM (*N*)	188.13 ± 93.33	0.629*	0.755*
Mean velocity – 30% 1RM (m/s)	1.18 ± 0.29	0.727*	0.790*
Peak power – 60% 1RM (W)	412.76 ± 314.00	0.678*	0.846*
Mean acceleration – 60% 1RM (m/s^2^)	2.24 ± 0.52	0.650*	0.510
Mean force – 60% 1RM (*N*)	385.21 ± 212.35	0.678*	0.825*
Mean velocity – 60% 1RM (m/s)	0.69 ± 0.12	0.653*	0.544
Peak power – 90% 1RM (W)	265.58 ± 170.86	0.734*	0.818*
Mean acceleration – 90% 1RM (m/s^2^)	0.38 ± 0.40	0.420	0.574
Mean force – 90% 1RM (*N*)	577.75 ± 305.82	0.678*	0.825*
Mean velocity – 90% 1RM (m/s)	0.27 ± 0.14	0.446	0.632*

* *p* ≤ 0.05.

Regarding the Medicine Ball (MB) throw test (Table 3), the estimated power generated showed a strong, significant, and negative correlation with both ST and JT. Conversely, the absolute distance of the MB throw did not show a significant correlation with ball velocity.

**Table 3:** Descriptive and correlation data for the medicine ball throw test.

Variable	Median ± IQR	Spearman's ρ (ST)	Spearman's ρ (JT)
Distance (m)	5.40 ± 0.59	−0.357	−0.245
Power (W)	730.88 ± 182.65	−0.692*	−0.643*

* *p* ≤ 0.05.

For the BPU test variations (Table 4), the most significant results emerged from the SCP. Specifically, relative power (W/kg) during the SCP showed a very robust correlation with JT velocity, representing one of the strongest associations between field tests and specific handball performance. The variations performed from a kneeling position did not yield significant correlations with throwing velocity. In the Drop-Fall Push-up variation, only absolute and relative power values demonstrated strong correlations with JT.

**Table 4:** Descriptive and correlation data for ballistic push-up (BPU) variables.

Variable	Median ± IQR	Spearman's ρ (ST)	Spearman's ρ (JT)
SCP height (cm)	8.59 ± 4.85	0.434	0.650*
SCP power (W)	1,717.04 ± 1,181.08	0.601*	0.671*
SCP relative power (W/kg)	24.85 ± 8.98	0.587*	0.734*
KCP height (cm)	14.35 ± 12.31	0.126	0.287
KCP power (W)	2,228.93 ± 1,508.28	0.455	0.566
KCP relative power (W/kg)	31.59 ± 12.61	0.098	0.245
DFP height (cm)	21.02 ± 14.68	0.357	0.490
DFP power (W)	2,182.45 ± 2,087.84	0.524	0.643*
DFP relative power (W/kg)	33.01 ± 15.04	0.476	0.608*

* *p* ≤ 0.05.

Lower-limb power assessed via the CMJ test (Table 5) revealed a strong and significant correlation between jump power output and JT velocity. Notably, this relationship was specific to the JT, as no significant association was found between lower-limb power and the ST.

**Table 5:** Descriptive and correlation data for CMJ.

Variable	Median ± IQR	Spearman's ρ (ST)	Spearman's ρ (JT)
Jump height (cm)	32.63 ± 11.84	0.420	0.629*
Power (W)	3,326.24 ± 1,499.91	0.573	0.748*
Relative power (W/kg)	44.45 ± 10.76	0.357	0.573

* *p* ≤ 0.05.

Finally, the descriptive data for the throwing tests (Table 6) indicated that the JT with progression was, on average, faster than the supported throw. A nearly perfect and significant correlation was observed between the velocities of the two throwing techniques.

**Table 6:** Descriptive data and correlation between throwing velocities.

Variable	Median ± IQR
Standing throw (ST, m/s)	21.11 ± 3.46
Jump throw (JT, m/s)	22.62 ± 3.97
Spearman's ρ	0.916*

* *p* ≤ 0.01.

## 4 Discussion

The primary objective of this study was to investigate the relationship between various upper-limb and lower-limb strength and power assessments and ball velocity in two distinct handball throwing techniques. Our findings partially confirm the initial hypothesis, demonstrating robust associations between physical performance measures and ball velocity. Notably, power-related variables showed consistent correlations with throwing performance, particularly in the JT. Furthermore, maximal strength emerged as a critical component for understanding overall performance.

Maximal dynamic strength, assessed via the 1RM bench press, exhibited a very strong correlation with JT (*ρ* = 0.842) and a strong correlation with ST (*ρ* **=** 0.688). These results align with seminal studies, such as Hoff and Almåsbakk (12), which demonstrated that increases in maximal bench press strength are associated with higher throwing velocities following training interventions. Similarly, Debanne et al. (14) suggest that while maximal strength is not the sole determinant, it constitutes a foundational capacity upon which power and movement velocity are developed. Our data also support the theory by Marques et al. (5) that dynamic, multi-joint assessments tend to yield higher correlations with throwing velocity compared to isometric or single-joint tests.

The analysis of submaximal bench press loads provided nuanced insights into how load intensity reflects the association with specific motor patterns. At 30% of 1RM, where movement velocity is maximized, we observed strong correlations for both ST and JT, particularly regarding mean acceleration (ST: *ρ* = 0.748; JT: *ρ* = 0.755) and mean velocity (ST: *ρ* = 0.727; JT: *ρ* = 0.790). This suggests a high degree of mechanical transfer between the ballistic bench press and the throwing motion at lower resistances. Interestingly, the 60% 1RM intensity yielded the highest correlation observed in the study for JT (peak power: *ρ* = 0.846), highlighting the relevance of this specific intensity for explosive actions. In contrast, at high intensities (90% 1RM), correlations remained strong for JT (*ρ* = 0.818 for peak power) but were less pronounced for ST.

This pattern suggests a functional distinction between the two throwing techniques across the force-velocity curve. The JT appears to be more dependent on force and power production under higher intensities, as evidenced by its stronger correlations with 1RM, 60%, and 90% loads. Conversely, the ST demonstrated higher correlations with lower submaximal loads (30% 1RM), indicating a greater functional similarity to high-velocity, low-resistance conditions. These findings could suggest that the jumping throw is more closely associated with the force-dominant portion of the force-velocity curve, while the standing throw benefits more from the high-velocity end of the spectrum where acceleration is maximized.

In contrast to the existing literature, our results for the MB throw revealed significant negative correlations with both ST (*ρ* = −0.692) and JT (*ρ* = −0.643), whereas previous studies have typically reported robust positive correlations (14, 15). A likely explanation for this discrepancy is the protocol adopted in the present study, in which participants performed the throw from a seated position with dorsal support, reducing the biomechanical similarity to the handball throwing kinetic chain.

The findings regarding the BPU are particularly noteworthy. Relative power during the SCP showed a strong correlation with ST (*ρ* = 0.587) and an even stronger correlation with JT (*ρ* = 0.734). These findings suggest that the BPU, which uses body mass as the external resistance, may represent a practical and accessible assessment of upper-limb explosive performance in handball players. To the best of our knowledge, this is the first study to report significant associations between BPU performance and handball throwing velocity. However, because the BPU relies on body mass as the external resistance, differences in body composition may influence the mechanical outputs obtained during the test. Therefore, body composition should be considered when interpreting the observed correlations, and future studies should further investigate its influence on the relationship between BPU performance and throwing velocity.

Finally, lower-limb power assessed via the CMJ showed a strong correlation specifically with the jumping throw (*ρ* = 0.748), but not with the ST. This finding reinforces the role of the lower limbs in technical gestures that involve jumping and the subsequent transfer of energy through the kinetic chain (28).

However, as a cross-sectional study with a limited sample size, these results should be considered a starting point for understanding the complex relationships between strength, power, and throwing performance. The sample included an equal number of male and female participants; however, the small number of participants within each sex subgroup precluded reliable sex-specific analyses. Therefore, data from both sexes were pooled for the present analyses, and the observed associations should be interpreted as general relationships rather than sex-specific effects. Future studies should include larger samples that allow for sex-specific analyses and validation of these associations across males and females and different competitive levels. Furthermore, longitudinal studies are essential to establish causality and determine whether specific training interventions at different bench press intensities can effectively enhance ball velocity.

## 5 Conclusion

The results of this study indicate that bench press-derived measures were significantly associated with ball velocity in Brazilian university handball players, suggesting that these assessments may reflect physical qualities related to throwing performance. Our findings reveal a specific functional pattern: the JT is more closely associated with strength and power produced at moderate and high intensities, whereas the ST shows a greater dependence on capacities executed at higher velocities with lower relative force requirements. Collectively, these results suggest that the bench press, evaluated across different intensities, may represent a useful functional indicator for monitoring performance and has strong applicability in strength and conditioning programs. In conclusion, these findings underscore the relevance of practical functional indicators, such as bench press assessments, for monitoring and guiding the training of university athletes. These findings may assist practitioners in monitoring physical performance; however, further longitudinal and intervention studies are required before these measures can be recommended for training prescription or predictive purposes. Future studies should investigate whether predictive models integrating physical, coordinative, and kinematic variables can improve the understanding of throwing performance.

## Data Availability

The raw data supporting the conclusions of this article will be made available by the authors, without undue reservation.
